# Genitourinary involvement in Klippel–Trénaunay syndrome: pathophysiology, evaluation, and management

**DOI:** 10.3389/fsurg.2025.1532509

**Published:** 2025-09-03

**Authors:** Weilong Lin, Chong Xie, Huaijie Wang, Weijia Yang, Peihua Wang, Zhengtuan Guo

**Affiliations:** Department of Pediatric Surgery and Vascular Anomalies, Xi’an International Medical Center Hospital, Xi’an, China

**Keywords:** Klippel–Trénaunay syndrome, uterus, bladder, dysmenorrhea, hematuria, menorrhagia, bleeding, pelvic pain

## Abstract

Klippel–Trénaunay syndrome (KTS) typically involves a combination of capillary, lymphatic, and venous malformations of the lower extremities. Genitourinary involvement is frequently observed in patients with KTS. Bleeding and pelvic pain are the most common complications. This condition has been increasingly reported in recent years. However, most authors have only depicted extreme presentations or various combinations of clinical findings. The underlying pathophysiology of genitourinary involvement in KTS remains unclear. Genitourinary involvement in female patients with KTS has a more complicated pathophysiology than that in male patients with KTS. After understanding its pathophysiology, some patients can be successfully managed by using a staged plan. Therefore, recognizing the pathophysiology of genitourinary involvement is necessary for practitioners to evaluate and determine adequate management. Owing to the complexity and rarity of this condition, a prospective controlled study involving a large cohort of patients is impossible. Based on a literature review and our practice, we discuss the pathophysiology, evaluation, and treatment strategies for genitourinary involvement in KTS.

## Introduction

Klippel–Trénaunay syndrome (KTS) is a rare congenital disorder, characterized by combination of capillary-lymphatic-venous malformations (1). It typically affects the lower extremities unilaterally and occasionally involves the upper or bilateral lower extremities. It can also affect the pelvic organs, abdomen, or trunk (1–3). Gastrointestinal and genitourinary (GU) involvement is common, with an incidence of more than 30% in patients (1, 2, 4). Gastrointestinal and GU involvement can be asymptomatic and can manifest as recurrent hemorrhage of the affected organs, ranging from occult to massive and life-threatening blood loss (1, 2, 4–6). GU involvement usually manifests as hematuria, dysmenorrhea, and menorrhagia (2, 4). Women with KTS and uterine involvement are at significant risk of severe postpartum hemorrhage (7, 8). However, these presentations remain challenging and require multidisciplinary management. Under these conditions, venous malformations (VM) infiltrate and surround the walls of the GU system, causing pain and hemorrhage. The management of hematuria and menorrhagia is conservative but dysmenorrhea in KTS patients can be treated pharmacologically (4). However, if problematic hemorrhage and dysmenorrhea develop, invasive treatments for the bleeding sites or affected organs are preferred, including surgical resection of the involved organ (4, 9–11), interventional embolization (12, 13), and sclerotherapy (14, 15).

Many patients with KTS with GU involvement have been reported in the literature; however, most authors have only depicted extreme manifestations or illustrated various interesting combinations of signs, symptoms, laboratory tests, images, and complications of KTS (5, 6, 16–52). Owing to the complexity, rarity, and under-recognized pathophysiology of GU involvement in KTS, an integrated management strategy for various treatments has not been established. No recommendations exist for the management of GU involvement in patients with KTS.

**Figure 1:**
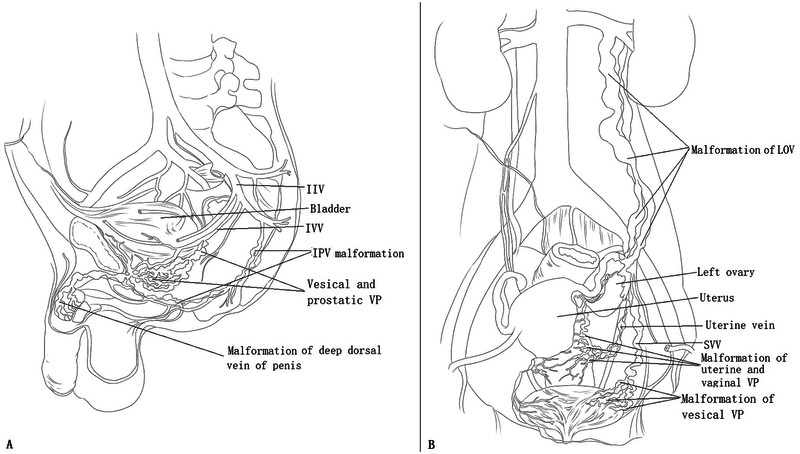
The principal pathophysiologies of genitourinary (GU) involvement in male and female patients with Klippel–Trénaunay syndrome (KTS). **(A)** In male patients with KTS with GU involvement, tributaries of the internal iliac vein (IIV) system are affected, including venous malformation (VM) of the vesical and prostatic venous plexus and the internal pudendal vein. The GU tract and circumferential VMs primarily drain into the inferior vesical vein and internal pudendal vein and then into the IIV. Thus, GU bleeding originates from VM of the tributaries of the IIV. VM of the deep dorsal vein of the penis usually manifests as dorsal phlebectasia or as a dorsal venous mass but does not cause bleeding. **(B)** In most female patients with KTS with GU involvement, only the tributaries of the IIV system are affected, including VM of the vesical, uterine, and vaginal venous plexuses, and the uterine vein. These VMs primarily drain into the superior vesical vein and uterine vein and then into the IIV. Thus, GU bleeding originates from the VM of the tributaries of the IIV. The left ovarian vein (LOV) is rarely involved and manifests as avalvulia, dilation, and reflux. In this condition, the LOV and vesical, uterine, and vaginal venous plexuses are intercommunicated. The LOV serves as a draining vein and is a possible cause of bleeding.

GU involvement in female patients has a more complicated pathophysiology than that of male patients with KTS (Figure 1). KTS can also affect the left ovary and its venous system. Of note, malformation and reflux of the uterine veins can also cause noncyclic pelvic pain and dysmenorrhea (53). Practitioners must be aware of the underlying pathophysiology of GU involvement in patients with KTS to improve their management. These vein malformations and reflux have been under-recognized in patients with KTS with pelvic involvement (2). Factors contributing to this lack of recognition include the rarity and complexity of pelvic VM in KTS, the challenge of distinguishing pelvic involvement in KTS from common pelvic venous disorders in adult women, the absence of validated diagnostic modalities and condition-specific treatment approaches, and limited reports on effective treatments. MRI can easily differentiate the anatomical differences of pelvic veins from veins in KTS (1). In a recent paper (1), we comprehensively discussed and reviewed the pathophysiology, evaluation, and management of gastrointestinal involvement in KTS. In this paper, we review and discuss its pathophysiology and propose an evaluation and treatment strategy that can help manage KTS with GU involvement.

## GU involvement in KTS in male patients

### Pathophysiology

In the current literature and in our practice, GU involvement in male patients primarily refers to bleeding from vascular malformations of the urethra and bladder (1, 4–6, 13, 16, 19, 27, 33, 35, 39, 40, 54–58). Confirmed hematuria of the ureter or kidney has rarely been reported and has not been encountered in our center.

In our previous study (2), avalvulia and reflux of the internal iliac vein (IIV) were identified in patients with KTS with pelvic involvement. The IIV, gluteal veins, and marginal veins comprise a valveless malformed reflux system (2). IIV reflux leads to congestion and focal venous hypertension of the pelvic organs, including the rectum, bladder, prostate, and urethra (2). VM develops slowly with age; therefore, chronic hematuria may manifest when a vein with submucosal reticular phlebectasia ruptures. The rupture of the vesical venous plexus causes painless hematuria. Rupture of the prostatic venous plexus can lead to bleeding from the prostate and urethra. These venous plexuses drain into the IIV through the inferior vesical vein and/or the internal pudendal vein (Figure 1).

Reflux or stagnant flow in a VM can predispose patients to thrombosis and thrombus propagation, which may trigger localized intravascular coagulopathy (LIC) (1, 59, 60). LIC is common in VM and features elevated D-dimer levels, low fibrinogen levels, and variable platelet counts (59, 60). Once the VM ruptures, bleeding rarely resolves spontaneously, but manifests as intermittent or persistent bleeding. The effects of LIC may have an important role in causing localized bleeding and/or thrombosis (59, 60). Anticoagulants such as rivaroxaban and low-molecular-weight heparin can be used to treat this localized coagulopathy (59, 60).

### Evaluation

Phlebectasia, VM, or capillary malformation of the scrotum/perineum usually exists in patients with KTS with GU involvement (24, 61). Intrascrotal masses of VM and lymphatic malformation can also exist, usually with little or no clinical significance (24). The pelvis is routinely evaluated in patients with KTS and GU manifestations by using magnetic resonance imaging (MRI) without contrast. If serial T2-weighted axial MRI scans of the pelvis revealed that the IIV had a higher fluid signal than that of the contralateral normal vein, we considered that IIV reflux was documented (2). In patients evaluated by using magnetic resonance venography (MRV), a significantly dilated IIV was considered to indicate IIV reflux (2). MRI can reveal IIV dilation, reflux, plexus VM, and venous incompetence (1, 2).

**Figure 2:**
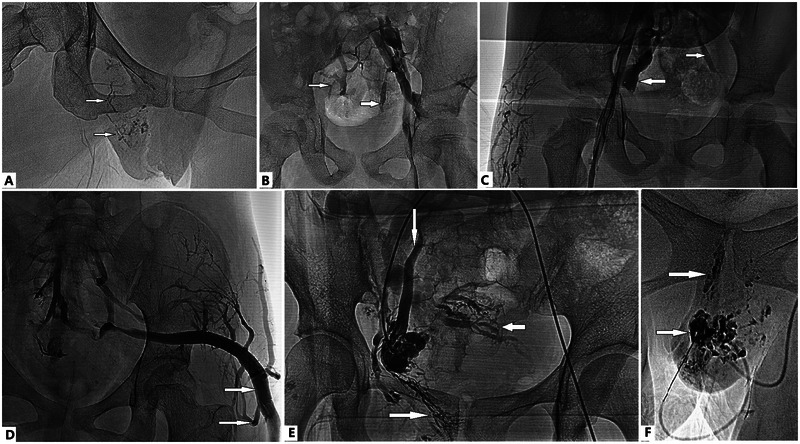
Anomalous venous flow in Klippel–Trénaunay syndrome (KTS) with genitourinary (GU) involvement. **(A)** Stagnant flow in dilated veins of the labia, vaginal wall, and uterus (arrows). **(B)** Centrifugal flow (i.e., reflux) of the internal iliac vein (IIV) from the common or external iliac vein (lower arrow) and active flow from the large vein into the smaller tributaries (upper arrow). **(C)** Flow of the IIV (left arrow) indicates and intercommunicates with tributaries of the contralateral IIV (right arrow) through the retroperitoneal venous plexus. **(B,D)** The large marginal superior gluteal vein is valveless, actively flows into smaller tributaries (arrows), and drains bilaterally into the IIV. **(E)** The patient is in the prone position, and the right popliteal vein is accessed. Transcatheter venography of the uterine VM through the IIV shows the flow draining into the vaginal veins (rightward arrow), the left ovarian vein (downward arrow), and its tributaries (leftward arrow). **(F)** Stagnant flow in spongiform VMs of the labia and vaginal wall (arrows).

We do not define malformative veins as dilated veins of > a certain millimeter in diameter. Phlebography is required to confirm MRI findings (2). Computed tomography is not routinely used to evaluate GU involvement in KTS. In our experience with MRI studies and complete phlebography of more than 500 patients with KTS, we defined pelvic VM in terms of venous hemodynamics rather than vein size. Anomalous venous flow includes stagnant flow, centrifugal flow (i.e., reflux), active flow from large veins into smaller tributaries, flow in persistent embryonic veins, venous drainage through extra-anatomic pathways, and flow in spongiform VMs (Figure 2). For example, when using our modified phlebography method or diversion phlebography (2), if free reflux occurred from the external or common iliac vein down into the IIV, continued into the rectal, vesical and/or prostatic venous plexus, and drained through the contralateral IIV, these findings were unified concepts of pelvic venous flow disorder in adult women (53), we considered these as pelvic involvement in KTS (1, 2).

Although the symptoms and venous malformation component can be determined by taking a careful history and performing comprehensive physical, ultrasound, and MRI examinations, complete phlebography is necessary to fully assess the pelvic hemodynamics in these patients and to determine the pathophysiologic component. Complete phlebography is also important and helpful in evaluating possible bleeding sites, identifying the responsible veins, and planning an interventional approach. Imaging of the femoral vein, marginal vein, gluteal veins, common iliac vein, external iliac vein, internal iliac vein, left gonadal vein, left renal vein, and pelvic venous plexus is required.

### Management

Bleeding management is initially conservative, and includes LIC management, reducing thrombotic risk and hence the averting of this stage by anticoagulant use, blood transfusions, iron supplements, sirolimus, and stool softeners (1, 4, 62). However, GU VM with clinically significant bleeding usually requires invasive management, including cauterization (4), embolization (12, 13), and surgical excision of the bleeding site (4, 9, 12, 54, 63, 64).

**Figure 3:**
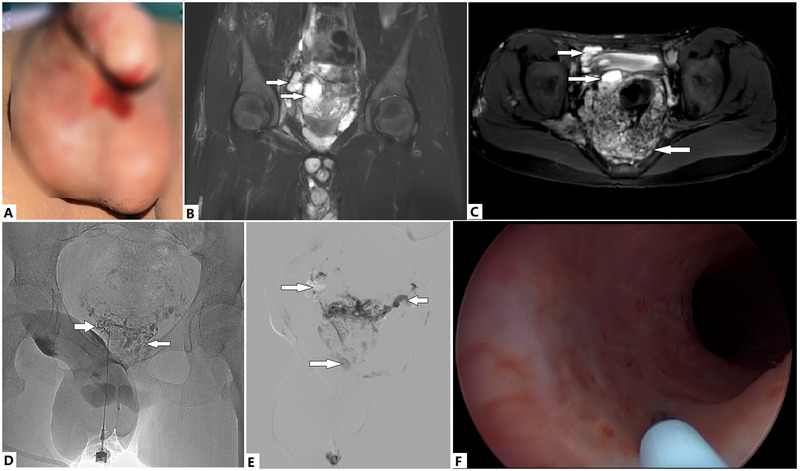
Evaluation and treatment of a 13-year-old boy with Klippel–Trénaunay syndrome (KTS) with genitourinary (GU) bleeding. At birth, the boy was diagnosed with KTS of the right lower extremity. **(A)** Recurrent hematuria and urethral bleeding have occurred in recent months, which have led to severe anemia and required repeated blood transfusions, despite his having undergone multiple medical treatments. **(B)** Venous malformations (VMs) of the prostatic and vesical venous plexuses, as revealed by T2 magnetic resonance imaging (T2 MRI) sequences (arrows). An intrascrotal VM is also visible. **(C)** A circumferential VM is encroaching on the vesical wall (upper rightward arrow). A cystic VM of the prostatic venous plexus is visible (lower rightward arrow). The rectal VM is also notable (left arrow). **(D)** VMs are directly punctured at the perineum, and vesical VM (right arrow) and prostatic VM are visible (left arrow). **(E)** Cystic VM of the prostatic venous plexus on MRI, which was confirmed with venography (lower right arrow). Ethanol and foam polidocanol were sequentially injected via puncture. These VMs (rightward arrows) drain into the contralateral inferior vesical vein (leftward arrow) through the malformed prostatic venous plexus. **(F)** Urethral bleeding is successfully managed with cystoscopic injection of ethanol at the bleeding sites. Hematuria and urethral bleeding completely resolved during the follow-up period of >1 year.

Sclerotherapy has recently become a popular management approach. On account these veins are drainage tributaries of the IIV, and bleeding of these venous plexuses is primarily from IIV reflux, two methods exist to approach these venous plexuses: direct puncture and trans-IIV access (Figure 3) (2). Ethanol-based sclerotherapy is the preferred treatment. Cystoscopic injection of ethanol at the bleeding sites and surgical resection of the bleeding site can also be considered if conservative treatments fail (Figure 3).

Intrascrotal masses of VMs and lymphatic malformations usually have little or no clinical significance, and therefore do not commonly require intervention (24). In rare cases, surgical removal is indicated for prevention of thrombosis in left renal vein or massive lesions after sclerotherapy failure.

Gross bleeding from malformations of the external urethral meatus may be seen in KTS if they are intertwined with engorge capillaries. Bleeding can be managed by the direct injection of bleomycin and topical sirolimus (65). Ethanol can cause scarring and partial necrosis of the glans penis; therefore, caution should be exercised in this condition.

## GU involvement in KTS in female patients

### Pathophysiology

**Figure 4:**
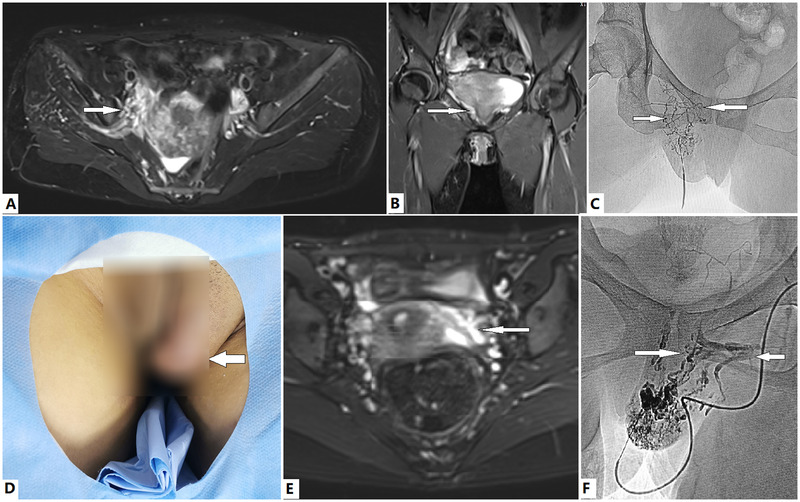
Evaluation and treatment of dysmenorrhea via direct puncture in female patients with Klippel–Trénaunay syndrome (KTS). **(A–C)** A 37-year old woman with KTS presented with progressive dysmenorrhea for several years. Dilated tributaries of the internal iliac vein, including the uterine veins, are identified by the T2 magnetic resonance imaging (T2 MRI) sequence **(A,B, arrows)**. The malformed labial, vaginal, and uterine veins **(C, arrows)** were venographed, embolized, and sclerosed with ethanol via direct puncture of the dilated labial veins. Dysmenorrhea was successfully managed after two treatment sessions. **(D–F)** A 16-year old girl with KTS presented with progressive dysmenorrhea since menarche. The labial venous malformation (VM) is visible **(D, arrow)**. Dilated incompetent uterine veins are revealed by T2 MRI sequences **(E, arrow)**. A typical fluid signal in the lumen indicates flow stagnation and/or reflux. The labial VM, vaginal veins **(F, rightward arrow)**, and incompetent uterine veins **(F, leftward arrow)** are venographically embolized and sclerosed with ethanol via direct puncture of the labial VM. Complete relief of dysmenorrhea was obtained after one session of treatment. However, the labial VM requires further management.

**Figure 5:**
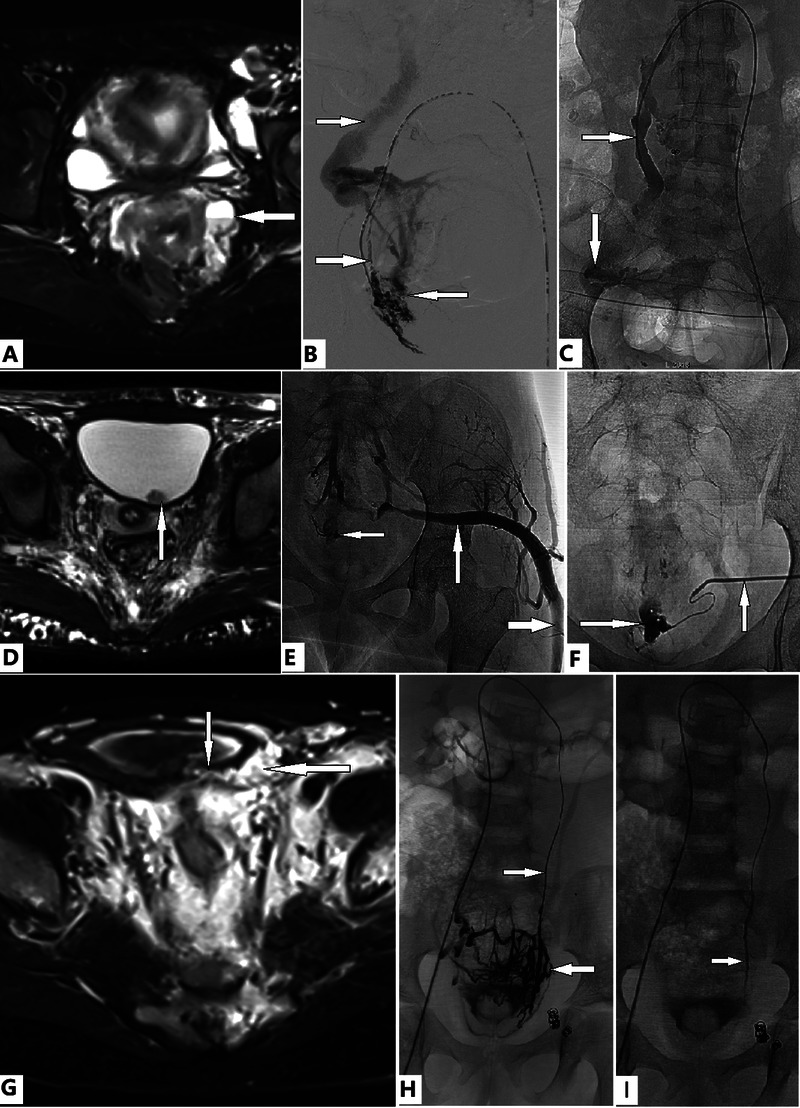
Evaluation and transcatheter treatment of genitourinary (GU) involvement in female patients with Klippel–Trénaunay syndrome (KTS). **(A–C)** A 38-year old woman with KTS presented with dysmenorrhea and severe anemia due to menorrhagia for years, which required multiple medications and repeated blood transfusions. T2 Magnetic resonance imaging (T2 MRI) reveals the ovary **(A, arrow)** and uterine venous malformation (VM). She is in the prone position. Transcatheter uterine venography reveals VM of the uterus and ovary **(B, leftward arrow)**, dual draining into the uterine veins **(B, lower rightward arrow)**, and the left ovarian vein (LOV), which is significantly dilated and incompetent **(B, upper rightward arrow)**. The uterine VM was managed with transcatheter ethanol and foam polidocanol. Treatment of the ovarian VM **(C, downward arrow)** through the LOV **(C, rightward arrow)** was performed in the same fashion. The patient's anemia and menorrhagia resolved completely. Complete relief from dysmenorrhea was obtained after two sessions of treatment. **(D–F)** A 14-year old girl with bilateral KTS presented with severe anemia due to persistent gross hematuria. Bladder bleeding is identified on magnetic resonance imaging **(D, arrow)**. The active bleeding site is identified by venography **(E, leftward arrow)**. The marginal superior gluteal venous channel **(E, rightward and upward arrows)** is large and connected to the bleeding VM of the bladder **(E, leftward arrow; F, rightward arrow)**. Transmicrocatheter management through the marginal superior gluteal venous channel **(F, upward arrow)** is performed in the same fashion, as described previously. Anemia and hematuria completely resolved during follow-up. **(G–I)** A 5-year old girl with KTS presented with moderate anemia due to intermittent gross hematuria. T2 MRI revealed that the bladder wall **(G, downward arrow)** was infiltrated by a VM **(G, leftward arrow)**. She had previously undergone transcatheter coil embolization and sclerotherapy of the left IIV; however, gross hematuria persisted. Dilation of the LOV **(H and I, rightward arrows)** is detected on imaging. Transmicrocatheter management through the left ovarian vein (LOV) was performed by using ethanol. The venous plexuses of the ovary and bladder are significantly intercommunicated **(H, leftward arrow)** and ablated with ethanol **(I)**. The anemia and hematuria resolved completely after treatment.

The pathophysiology of GU involvement is more complicated in women than in men with KTS (Figure 1). Men and women can experience hematuria. However, in female patients, dysmenorrhea and menorrhagia can also occur when VM affects the uterus and the vagina (Figure 4) (2, 12, 20, 23, 32, 36, 37, 43, 52, 66). In women with KTS with GU involvement, a valveless IIV system has a broad communication network and provides a large capacitance to accommodate hemodynamic alterations. The left renal hilum, pelvic visceral and parietal veins, and superficial extrapelvic veins serve as venous reservoirs (67). At the pelvic escape points, these reservoirs communicate with the superficial extrapelvic veins in the thighs and the perineum through the pelvic floor. These veins communicate with the left renal hilum through the left ovarian vein (67). Congenital pelvic VMs in KTS result in stagnant pelvic venous flow, hypertension, and congestion (2), which can be transmitted to the adjacent venous plexus, thereby leading to associated symptoms and phlebectasia. The etiologies underlying dysmenorrhea and menorrhagia in patients with KTS with GU involvement are complicated. Our previous study (2) demonstrated that IIV reflux may be associated with GU bleeding, dysmenorrhea, and menorrhagia in female patients with lower extremity KTS. Pelvic pain can also be caused by reflux of the left ovarian vein. In practice, the coexistence of reflux and IIV has been observed in some patients with KTS and dysmenorrhea (Figure 5). Pain relief can be achieved after ablation of reflux (2). In some patients, dysmenorrhea may be exclusively caused by incompetence of the uterine veins (Figure 4). In patients with KTS with dysmenorrhea and/or menorrhagia, the etiologies include IIV reflux, left ovarian vein reflux, uterine VM, or both.

Mechanical venous hypertension may be caused by outflow obstruction of the gravid uterus. Pregnancy may also precipitate and worsen existing pelvic VM because progesterone and estrogen are at extreme physiological levels during pregnancy, which can increase the circulating blood volume and worsen focal venous hypertension (7, 8, 49, 52, 68, 69). Therefore, some patients show symptom progression during pregnancy (68). On account of focal venous hypertension and possible LIC, rupture of the vesical venous plexus, vaginal plexus, and uterine venous plexus causes hematuria, abnormal colporrhagia, and menorrhagia, respectively.

### Evaluation

Phlebectasia, VM, or capillary malformation of the external genitalia/perineum usually exist in female patients with KTS with pelvic involvement and often affect the labia (Figure 4) (68, 70, 71). The IIV and vesical, vaginal, and uterine venous plexuses require a full assessment of venous hemodynamics by using MRI and confirmative phlebography. Imaging of the left ovarian vein is also required, in addition to imaging of the femoral vein, marginal vein, gluteal veins, common iliac vein, external iliac vein, and IIVs. Phlebectasia/VM of the external genitalia/perineum can be punctured directly to perform phlebography of the pelvic veins (Figure 4).

### Management

If conservative treatment fails, sclerotherapy is the most commonly used treatment strategy. The aforementioned pathophysiology of hematuria and abnormal colporrhagia and menorrhagia and interventional radiological approaches to the pelvic venous plexuses include direct puncture, trans-IIV access, and trans-left ovarian vein access (Figure 5). If phlebectasia/VMs of the external genitalia/perineum are visible and accessible, direct puncture of these veins can be used to perform ethanol-based sclerotherapy (Figure 4). Because the marginal vein often drains into the IIV via the inferior and superior gluteal veins, these veins can also be accessed and used for transcatheter intervention through the marginal vein (Figure 5). We preferred administering sclerotherapy with a combination of ethanol and foam polidocanol. Cystoscopic injection of ethanol at the bleeding sites, cauterization, and surgical resection of the bladder bleeding sites can also be considered. Hysterectomy is rarely required in patients with KTS with uterine involvement.

## Obstetric considerations for patients with KTS with uterine involvement

KTS has previously been considered a contraindication for pregnancy (72). Some considerations should be considered when planning a successful delivery in patients with KTS and uterine involvement. Owing to the rarity and complexity of KTS during pregnancy, most studies are case reports; each condition is variable, and standard care for delivery seems impossible.

The overall stagnant flow in the uterine and vaginal venous plexus may markedly progress during pregnancy; therefore, predelivery MRI of the pelvis and spine is required to guide anesthesia options (52, 73). If a VM is noted in the spinal canal, regional anesthesia for any surgery, including delivery, is unsuitable. Careful mapping of the dilated veins, performing a comprehensive examination of the patient to assess the veins' potential for expansion, and pelvic and spinal MRI are recommended (74). MRI can be used to identify the location of the VM and determine whether neuroaxial anesthesia can be performed (52, 73).

In the literature, cesarean section delivery has been described in more than one-half of pregnancies in patients with KTS, but a cross-sectional study (8) demonstrated that cesarean sections were performed significantly less frequently in the KTS group than in the reference population. In practice, physicians may prefer vaginal delivery to prevent bleeding-related complications caused by a VM close to the uterus (8). Midline and paramidline incisions can avoid dilated veins and reduce blood loss (7, 75). Therefore, the delivery method should be determined by an obstetric-led team, after weighing the individual's clinical risks and benefits.

Compared with population-based cohorts, patients with KTS have an increased risk of severe postpartum hemorrhage (8). Bleeding may occur because of rupture of a uterine VM and possible LIC in a large VM. Whether the use of prophylactic or therapeutic anticoagulants during the antenatal and postnatal periods is a risk factor for postpartum hemorrhage has not yet been determined (8). For LIC, anticoagulants can be used to improve focal consumptive coagulopathy in the VM (60).

Postpartum hemorrhage in patients with KTS with uterine involvement is from the pelvic VM, similar to the aforementioned pathophysiology; therefore, trans-IIV embolization is an alternative approach to stop bleeding. Owing to the venous origin of postpartum hemorrhage, limited treatment success using uterine artery and internal iliac artery embolization has been reported in the literature (7). However, the management of massive obstetric hemorrhages is individually dependent. Emergency hysterectomy is the most common surgical treatment for uncontrollable bleeding (7).

## Future perspectives

GU involvement with KTS remains a treatment challenge. Patients with KTS harbor a somatic mosaic mutation in the *PIK3CA* gene. Therefore, a positive response to direct phosphatidylinositol-3-kinase (PI3K) inhibitors can be expected in patients with problematic KTS (62). Alpelisib, a PI3K inhibitor, has been specifically approved for *PIK3CA*-related overgrowth spectrum to reduce overgrowth, vascular components, and other functional complications (76). In the future, PI3K inhibitors should be clinically trialed and evaluated with regard to their ability to manage GU complications of KTS such as bleeding and pain.

## Conclusion

KTS is a rare slow-flow condition associated with vascular malformation (i.e., capillary, lymphatic, and venous) and limb overgrowth. In the current literature and in our study, GU involvement in male patients with KTS primarily refers to hematuria, urethral bleeding, and intrascrotal venous-lymphatic malformation. In women with KTS, GU involvement refers to bleeding from vascular malformations of the urinary tract, uterus, and vagina and pelvic pain from malformations. Male patients have malformations and abnormal hemodynamics of the IIV system. Therefore, the venous system must be evaluated. Female patients have malformations and abnormal hemodynamics of two vein systems: the IIV and the left ovarian vein. Recognizing these pathophysiologies is necessary to comprehensively assess the underlying venous system and plan management.

This review discussed the pathophysiology of GU involvement in KTS and highlighted the evaluation and management approaches for underlying vascular malformations. The issues discussed in this paper may provide clinical references for practitioners. Despite our efforts to integrate the current knowledge regarding GU involvement in KTS, our analysis was based on a low level of evidence from a small number of patients, and the conclusions may be controversial.
